# Supplementary material for: Sustained Negative Mental Health Outcomes Among Healthcare Workers Over the First Year of the COVID-19 Pandemic: A Prospective Cohort Study
Source: Int J Public Health. 2022 Jun 17;67:1604553. doi: 10.3389/ijph.2022.1604553 (PMC9266625; doi:10.3389/ijph.2022.1604553)
Supplement: Supplementary file 1 [file DataSheet1.PDF]

Sustained negative mental health outcomes among healthcare workers over the first year of the COVID-19 pandemic: a prospective cohort study

Supplementary material

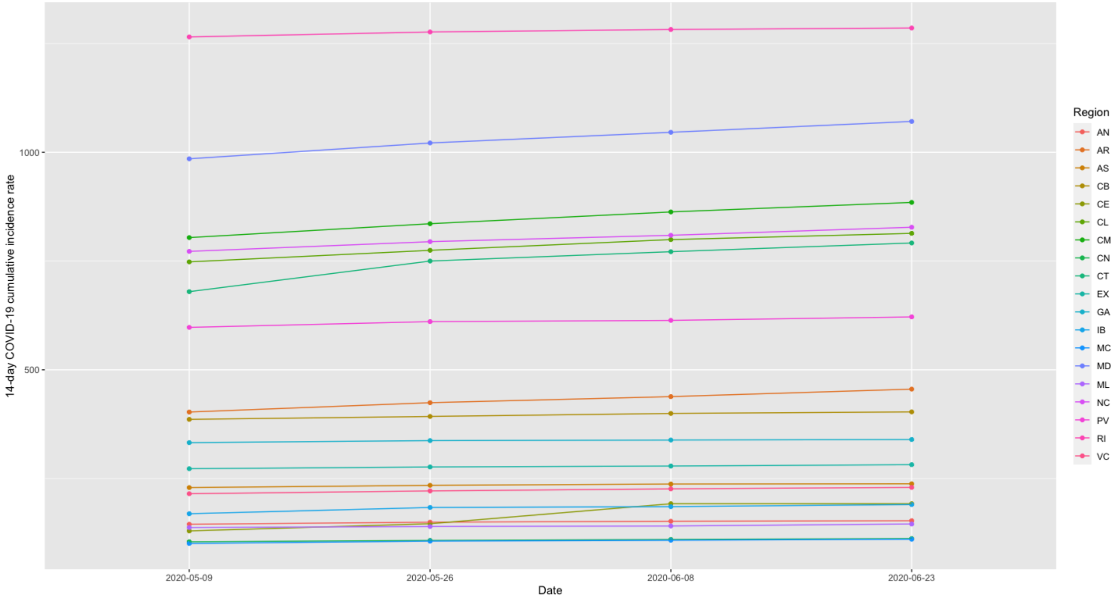

Supplementary Figure 1. 14-day cumulative incidence by region at 2, 4, 6 and 8 weeks after the study onset. AN = Andalucía, AR = Aragón, AS = Asturias, CB = Cantabria, CE = Ciudad Autónoma de Ceuta, CL = Castilla y León, CM = Castilla-La Mancha, CN = Canarias, CT = Cataluña, EX = Extremadura, GA = Galicia, IB = Islas Baleares, MC = Región de Murcia, ML = Ciudad Autónoma de Melilla, NC = Comunidad Foral de Navarra, PV = País Vasco, RI = La Rioja, VC = Comunidad Valenciana

[TheCOVID-19 HEalth caRe wOrkErS (HEROES) Study, Spain, 2021]

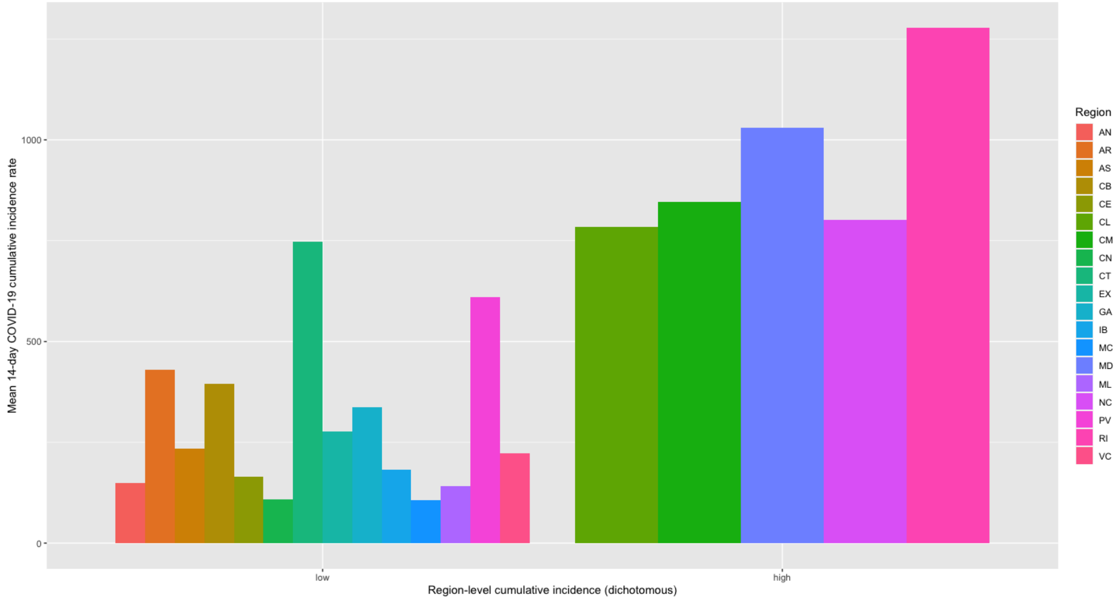

Supplementary Figure 2. 14-day cumulative incidence by region at 2, 4, 6 and 8 weeks after the study onset (bis). AN = Andalucía, AR = Aragón, AS = Asturias, CB = Cantabria, CE = Ciudad Autónoma de Ceuta, CL = Castilla y León, CM = Castilla-La Mancha, CN = Canarias, CT = Cataluña, EX = Extremadura, GA = Galicia, IB = Islas Baleares, MC = Región de Murcia, ML = Ciudad Autónoma de Melilla, NC = Comunidad Foral de Navarra, PV = País Vasco, RI = La Rioja, VC = Comunidad Valenciana
[The COVID-19 HEalth caRe wOrkErS (HEROES) Study, Spain, 2021]

Supplementary Table 1.
Sampling strategy across health and care facilities in the main study locations (Andalucía, Madrid, and Murcia) [The COVID-19 HEalth caRe wOrkErS (HEROES) Study, Spain, 2021]

Region	Recruitment unit	Type of unit	Sampling strategy
All	Sociedad Española de Medicina Preventiva, Salud Pública e Higiene (SEMPSPH)	Medical association	Representativeness approached by researcher; snowball sampling (text messages, emails)
All	Asociación Nacional de Psicólogos y Residentes (ANPIR)	Psychological association	Representativeness approached by researcher; snowball sampling (text messages, emails)
Cádiz	Hospital Universitario Puerto Real	General hospital	Representativeness approached by researcher
Granada	Hospital Regional Clínico Universitario San Cecilio	General hospital	Representativeness approached by researcher
Granada	Unidad de Gestión Clínica Almanjáyar	Outpatient specialty center	Representativeness approached by researcher
Granada	Unidad de Gestión Clínica Cartuja	Outpatient specialty center	Representativeness approached by researcher
Granada	Unidad de Gestión Clínica Gran Capitán	Outpatient specialty center	Representativeness approached by researcher
Huelva	Hospital Juan Ramón Jiménez	General hospital	Representativeness approached by researcher
Madrid	Colmenar Viejo Sur	Outpatient specialty center	Snowball sampling (text messages); billboard
Madrid	Hospital Universitario La Paz	General hospital	Pop-up alert embedded in electronic system; snowball sampling (text messages); billboard
Madrid	Oficina Regional de Salud Mental	Outpatient specialty center	Representativeness approached by researcher
Madrid	Hospital Universitario La Princesa	General hospital	Snowball sampling (text messages)
Madrid	Unión General de Trabajadores (UGT)	Labor union	Representativeness approached by researcher
Madrid	Comisiones Obreras (CCOO)	Labor union	Representativeness approached by researcher
Madrid	Sindicato de Enfermería (SATSE)	Labor union	Representativeness approached by researcher
Madrid	Asociación de Médicos y Titulados Superiores (AMYTS)	Labor union	Representativeness approached by researcher
Málaga	Unión General de Trabajadores (UGT)	Labor union	Representativeness approached by researcher
Málaga	Hospital Regional Universitario de Málaga	General hospital	Snowball sampling (text messages)
Málaga	Central Sindical Independiente y de Funcionarios (CSIF)	Labor union	Representativeness approached by researcher
Málaga	Comisiones Obreras (CCOO)	Labor union	Representativeness approached by researcher
Málaga	Hospital Universitario Virgen de la Victoria	General hospital	Snowball sampling (text messages)
Málaga	Sindicato de Enfermería (SATSE)	Labor union	Representativeness approached by researcher

Málaga	Sindicato de Técnicos Auxiliares de Enfermería (SAE)	Labor union	Representativeness approached by researcher
Málaga	Local non-hospital emergency departments (Málaga-Guadalhorce)	Non-hospital emergency department	Representativeness approached by researcher; snowball sampling (text messages, emails)
Málaga	Hospital Comarcal de la Axarquía	General hospital	Representativeness approached by researcher
Málaga	Hospital Comarcal Antequera	General hospital	Representativeness approached by researcher
Málaga	Medical Residents Coordination Office	Professional network	Representativeness approached by researcher
Málaga	Hospital San Juan de Dios	Private hospital	Representativeness approached by researcher
Málaga	Local health office (Norte de Málaga-Antequera)	Primary care center	Representativeness approached by researcher
Málaga	Local health office (Serranía de Málaga)	Primary care center	Representativeness approached by researcher
Málaga	Local health office (Este de Málaga-Axarquia)	Primary care center	Representativeness approached by researcher
Murcia	Hospital Clínico Universitario Virgen de la Arrixaca	General hospital	Representativeness approached by researcher; snowball sampling (text messages, emails)
Murcia	Hospital Los Arcos	General hospital	Representativeness approached by researcher; snowball sampling (text messages, emails)
Murcia	Oficina Regional de Salud Mental	Outpatient specialty center	Representativeness approached by researcher
Murcia	Instituto Murciano de Investigación Biomédica (IMIB)	Health research institute	Representativeness approached by researcher; snowball sampling (text messages, emails)
Murcia	Colegio de Médicos	Medical association	Representativeness approached by researcher
Murcia	Colegio de Enfermería	Nursing association	Representativeness approached by researcher
Murcia	Colegio de Farmacia	Pharmaceutical association	Representativeness approached by researcher
Murcia	Colegio de Fisioterapia	Physiotherapeutical association	Representativeness approached by researcher
Murcia	Comité Empresa (Sindicato)	Labor union	Representativeness approached by researcher
Murcia	Primary care network	Professional network	Representativeness approached by researcher
Sevilla	Hospital Universitario Virgen de Valme	General hospital	Representativeness approached by researcher
Sevilla	Área de Gestión Sanitaria Sur de Sevilla	Primary care center	Representativeness approached by researcher

Supplementary Table 2.
Response rates across recruitment units [The COVID-19 HEalth caRe wOrkErS (HEROES) Study, Spain, 2021]

Region	Recruitment unit	Type of unit	Response rate (%)
Spain	Grupo 5	Socio-community mental health centers	28.2<br>67.6 [2]
Madrid	Hospital Universitario La Paz	General hospital	
	Emergency Department (Children's hospital)		
	Nurses		3.1
	Nurse technicians		7.7
	Total		5.2
	Emergency Department (Adult hospital)		
	Nurses		6.7
	Nurse technicians		3.5
	Physicians		25.5
	Total		8.6
	Department of General Surgery (Adult hospital)		
	Nurses		19.6
	Nurse technicians		5.6
	Physicians		13.4
	Total		13.4
	ICUs (Adult hospital)		
	Nurses		10.9
	Nurse technicians		2.7
	Physicians		8.0
	Total		8.1
	ICU (Children's Hospital: nurses)		8.9
	Department of Anesthesiology (physicians)		4.2
	Department of Internal Medicine (physicians)		22.2
	Department of Gastroenterology (physicians)		6.1
	Department of Pneumology (physicians)		6.9
	Department of Neurology (physicians)		7.7
	Department of Mental Health		
	Psychiatrists		34.1
	Clinical psychologists		14.8
	Total		26.8
	Orderly team		26.1
Andalucía	FAISEM	Socio-community mental health centers	24.0
Badajoz	Talarrubias	Primary care center	90.0
Granada	Almanjayar	Primary care center	38.5
Granada	Cartuja	Primary care center	21.9
Granada	Gran Capitán	Primary care center	12.3
Málaga	Alameda Perchel	Primary care center	36.8
Málaga	Rincón de la Victoria	Primary care center	8.3
Málaga	Urgencias Distrito Hospital Clínico	Emergency Unit	14.6
Murcia	Los Barreros	Primary care center	68.2
Murcia	Isaac Peral	Primary care center	27.5
Murcia	San Andrés	Primary care center	16.7
Murcia	Mental health center of Cartagena	Mental health center	80.4
Murcia	Consejería de Salud (COVID-19 contact tracers)	Regional Health Office	81.3
Murcia	Consejería de Salud (others)	Regional Health Office	35.0
Murcia	Hospital Clínico Universitario Virgen de la Arrixaca	General hospital	
	Department of Pediatrics		100
	Department of Preventive Medicine		94.4
	Department of Oncology		5.7

Supplementary Table 3.

Psychological distress, depression symptoms, and posttraumatic stress disorder symptoms among all follow-up respondents (N = 1,807) [The COVID-19 HEalth caRe wOrkErS (HEROES) Study, Spain, 2021]

	Psychological distress (GHQ-12)		Depression symptoms (PHQ-9)		PTSD symptoms (PC-PTSD-5)	
	n (%) [a]	M (SD) [b]	n (%) [a]	M (SD) [b]	n (%) [a]	M (SD) [b]
Overall	881 (56)	3.8 (3.4)	326 (21)	6.3 (5.1)	781 (51)	2.6 (1.6)
Age group						
18-35	290 (59)	4 (3.3)	115 (25)	6.9 (4.8)	273 (60)	2.9 (1.5)
36-50	367 (56)	3.9 (3.5)	140 (22)	6.5 (5.2)	334 (53)	2.6 (1.6)
Over 50	204 (52)	3.6 (3.5)	64 (16)	5.6 (5.1)	157 (40)	2.2 (1.6)
Gender						
Male	166 (48)	3.3 (3.5)	45 (13)	5.3 (5.1)	133 (40)	2.1 (1.5)
Female	714 (58)	3.9 (3.4)	281 (23)	6.7 (5)	648 (55)	2.7 (1.6)
Educational level						
Primary studies	5 (36)	3 (3.7)	2 (13)	5 (6.6)	6 (40)	2.1 (1.7)
Secondary studies	176 (51)	3.7 (3.5)	75 (22)	6.6 (5.5)	181 (54)	2.7 (1.6)
University studies	698 (57)	3.9 (3.4)	249 (21)	6.3 (4.9)	591 (51)	2.5 (1.6)
Parental educational level						
Primary studies	267 (52)	3.6 (3.4)	96 (19)	6 (5)	245 (49)	2.5 (1.6)
Secondary studies	275 (58)	3.9 (3.3)	96 (20)	6.6 (4.8)	250 (55)	2.7 (1.5)
University studies	306 (56)	3.9 (3.5)	121 (23)	6.3 (5.1)	258 (50)	2.5 (1.6)
Type of job						
Physicians	233 (60)	4.2 (3.5)	82 (21)	6.5 (5.1)	183 (48)	2.4 (1.7)
Nurses	172 (58)	4.1 (3.6)	75 (27)	7 (5.1)	174 (64)	3 (1.5)
Health technicians [c]	44 (55)	4.1 (3.8)	24 (32)	8.1 (6.2)	48 (67)	3 (1.6)
Other HCWs [d]	131 (52)	3.2 (2.9)	42 (17)	5.6 (4.4)	117 (47)	2.4 (1.6)
Ancillary workers [e]	74 (51)	3.5 (3.5)	26 (18)	6.1 (5.3)	64 (45)	2.5 (1.6)
Residential support workers	179 (51)	3.6 (3.4)	60 (17)	5.9 (4.9)	163 (49)	2.5 (1.5)
Other	48 (63)	4.1 (3.2)	17 (22)	6.7 (5.1)	32 (44)	2.3 (1.3)

Note

All percentages are valid percentages

GHQ-12 = General Health Questionnaire – 12, PHQ-9 = Patient Health Questionnaire – 9, PTSD = posttraumatic stress disorder, PC-PTSD-5 = Primary Care PTSD Screen for the DSM-5, HCWs = healthcare workers, PPE = personal protective equipment

[a] Number of respondents screening positive for mental health problems (cutoffs: PHQ-9 > 9, GHQ-12 > 2, and PC-PTSD > 2)

[b] Means and standard deviations of the total scores of the PHQ-9 (range: 0-27), the GHQ-12 (range: 0-12), and the PC-PTSD-5 (range: 0-5)

[c] Health technicians include nurse, X-ray, or laboratory technicians, among others

[d] Other HCWs include clinical psychologists, physiotherapists, or biologists, among others

[e] Ancillary workers include security staff, drivers, administrative staff, or cleaning staff, among others

Supplementary Table 4.

Association between participants' sociodemographic characteristics and COVID-related exposures, measured at baseline, and the probability of screening positive for psychological distress, depressive symptoms, and PTSD symptoms, at follow-up (8 months) [The COVID-19 HEalth caRe wOrkErS (HEROES) Study, Spain, 2021]

	Psychological distress (GHQ-12)				Depression symptoms (PHQ-9)				PTSD symptoms (PC-PTSD-5)			
	Unadjusted		Adjusted		Unadjusted		Adjusted		Unadjusted		Adjusted	
	OR	95 percent CI	OR	95 percent CI	OR	95 percent CI	OR	95 percent CI	OR	95 percent CI	OR	95 percent CI
18-35 years old [ref: > 50] [a]	1.03	(0.67, 1.58)	0.94	(0.61, 1.46)	1.60	(0.94, 2.71)	1.35	(0.78, 2.33)	1.77	(1.15, 2.74)	1.58	(1.01, 2.46)
35-50 years old [ref: > 50] [a]	0.89	(0.6, 1.33)	0.84	(0.56, 1.26)	1.24	(0.75, 2.07)	1.15	(0.69, 1.93)	1.31	(0.87, 1.96)	1.23	(0.82, 1.86)
Female gender [ref: male] [b]	1.82	(1.2, 2.77)	1.77	(1.15, 2.72)	3.62	(1.75, 7.46)	3.21	(1.54, 6.69)	1.91	(1.24, 2.93)	1.73	(1.11, 2.7)
University studies [ref: primary/secondary] [c]	0.76	(0.46, 1.23)	0.76	(0.44, 1.3)	0.61	(0.36, 1.03)	0.58	(0.32, 1.05)	0.78	(0.48, 1.27)	0.78	(0.45, 1.33)
Frontline position [ref: no] [c]	0.92	(0.67, 1.28)	0.87	(0.61, 1.24)	0.92	(0.61, 1.38)	0.86	(0.54, 1.36)	1.68	(1.21, 2.35)	1.75	(1.2, 2.54)
Adequate access to PPE [ref: inadequate] [c]	0.61	(0.44, 0.85)	0.66	(0.47, 0.93)	0.72	(0.48, 1.09)	0.83	(0.54, 1.29)	0.58	(0.41, 0.81)	0.63	(0.44, 0.9)
Fear of getting infected [ref: none or low] [c]	1.32	(0.94, 1.86)	1.29	(0.9, 1.84)	1.19	(0.77, 1.83)	1.10	(0.69, 1.74)	1.79	(1.26, 2.53)	1.69	(1.16, 2.45)
Fear of infecting loved ones [ref: none or low] [c]	1.15	(0.77, 1.72)	1.20	(0.78, 1.85)	0.98	(0.59, 1.62)	0.88	(0.51, 1.52)	1.96	(1.3, 2.97)	2.16	(1.37, 3.4)

Note

GHQ-12 = General Health Questionnaire – 12 items, PHQ-9 = Patient Health Questionnaire – 9 items, PC-PTSD-5 = Primary Care PTSD Screen for DSM-5, OR = odds ratio, CI = confidence interval, PPE = personal protective equipment

[a] Adjusted for gender

[b] Adjusted for age

[c] Adjusted for age, gender, and region-level 14-day COVID-19 cumulative incidence (fixed factor)

Supplementary Table 5.

Association between participants' sociodemographic characteristics and COVID-related exposures, measured at baseline, and mental health outcomes' total scores (psychological distress, depressive symptoms, and PTSD symptoms), measured at follow-up (8 months) [The COVID-19 HEalth caRe wOrkErS (HEROES) Study, Spain, 2021]

	Psychological distress (GHQ-12)				Depression symptoms (PHQ-9)				PTSD symptoms (PC-PTSD-5)*			
	Unadjusted		Adjusted		Unadjusted		Adjusted		Unadjusted		Adjusted	
	B	95 percent CI	B	95 percent CI	B	95 percent CI	B	95 percent CI	B	95 percent CI	B	95 percent CI
Age in years [a]	0.00	(-0.03, 0.02)	0.01	(-0.01, 0.04)	-0.04	(-0.08, 0)	0.01	(-0.02, 0.04)	-0.02	(-0.04, -0.01)	-0.02	(-0.03, -0.01)
Female gender [b]	1.05	(0.32, 1.77)	0.32	(-0.37, 1.01)	2.14	(1.1, 3.18)	0.57	(-0.32, 1.46)	0.68	(0.34, 1.02)	0.31	(-0.02, 0.64)
Educational level [c]	-0.22	(-0.57, 0.12)	-0.29	(-0.63, 0.05)	-0.54	(-1.02, -0.05)	-0.36	(-0.79, 0.07)	-0.16	(-0.32, 0)	-0.23	(-0.38, -0.07)
Frontline position [c]	0.13	(-0.42, 0.69)	-0.31	(-0.88, 0.25)	0.08	(-0.72, 0.89)	-0.68	(-1.41, 0.04)	0.46	(0.2, 0.73)	0.33	(0.06, 0.6)
Adequate access to PPE [c]	-0.53	(-0.86, -0.2)	-0.20	(-0.52, 0.12)	-1.03	(-1.5, -0.56)	-0.41	(-0.81, 0)	-0.37	(-0.52, -0.21)	-0.21	(-0.36, -0.06)
Fear of getting infected [c]	0.25	(-0.13, 0.63)	-0.20	(-0.58, 0.18)	0.42	(-0.12, 0.96)	-0.30	(-0.78, 0.17)	0.46	(0.28, 0.64)	0.27	(0.09, 0.45)
Fear of infecting loved ones [c]	0.32	(-0.02, 0.66)	0.13	(-0.2, 0.46)	0.66	(0.17, 1.16)	0.03	(-0.4, 0.45)	0.50	(0.34, 0.65)	0.42	(0.27, 0.57)

Note

GHQ-12 = General Health Questionnaire – 12 items, PHQ-9 = Patient Health Questionnaire – 9 items, PC-PTSD-5 = Primary Care PTSD Screen for DSM-5, B = beta, CI = confidence interval, PPE = personal protective equipment

[a] Adjusted for gender and GHQ-12 / PHQ-9 baseline total score

[b] Adjusted for age and GHQ-12 / PHQ-9 baseline total score

[c] Adjusted for age, gender, GHQ-12 / PHQ-9 baseline total score, and region-level 14-day COVID-19 cumulative incidence (fixed factor)

* Baseline assessment did not include PC-PTSD-5. Models are corrected using GHQ-12 baseline total score instead

Supplementary Table 6.

Association between participants' sociodemographic characteristics and COVID-related exposures, measured at baseline, and the probability of screening positive for psychological distress, depressive symptoms, and PTSD symptoms, at follow-up (8 months) [The COVID-19 HEalth caRe wOrkErS (HEROES) Study, Spain, 2021]

	Psychological distress (GHQ-12)				Depression symptoms (PHQ-9)				PTSD symptoms (PC-PTSD-5)*			
	Unadjusted		Adjusted		Unadjusted		Adjusted		Unadjusted		Adjusted	
	OR	95 percent CI	OR	95 percent CI	OR	95 percent CI	OR	95 percent CI	OR	95 percent CI	OR	95 percent CI
18-35 years old [ref: > 50] [a]	1.03	(0.67, 1.58)	0.92	(0.57, 1.48)	1.60	(0.94, 2.71)	1.14	(0.61, 2.12)	1.77	(1.15, 2.74)	1.59	(0.97, 2.61)
35-50 years old [ref: > 50] [a]	0.89	(0.6, 1.33)	0.77	(0.49, 1.2)	1.24	(0.75, 2.07)	1.02	(0.56, 1.84)	1.31	(0.87, 1.96)	1.21	(0.76, 1.9)
Female [ref: male] [b]	1.82	(1.2, 2.77)	1.36	(0.85, 2.16)	3.62	(1.75, 7.46)	2.34	(1.08, 5.08)	1.91	(1.24, 2.93)	1.33	(0.82, 2.16)
University studies [ref: primary/secondary] [c]	0.76	(0.46, 1.23)	0.69	(0.37, 1.26)	0.61	(0.36, 1.03)	0.70	(0.35, 1.42)	0.78	(0.48, 1.27)	0.79	(0.43, 1.44)
Frontline position [ref: no] [c]	0.92	(0.67, 1.28)	0.82	(0.55, 1.2)	0.92	(0.61, 1.38)	0.78	(0.46, 1.31)	1.68	(1.21, 2.35)	1.71	(1.14, 2.58)
Adequate access to PPE [ref: inadequate] [c]	0.61	(0.44, 0.85)	0.71	(0.5, 1.03)	0.72	(0.48, 1.09)	0.84	(0.51, 1.38)	0.58	(0.41, 0.81)	0.71	(0.48, 1.05)
Fear of getting infected [ref: none or low] [c]	1.32	(0.94, 1.86)	1.05	(0.71, 1.54)	1.19	(0.77, 1.83)	1.00	(0.6, 1.66)	1.79	(1.26, 2.53)	1.18	(0.78, 1.79)
Fear of infecting loved ones [ref: none or low] [c]	1.15	(0.77, 1.72)	1.05	(0.66, 1.68)	0.98	(0.59, 1.62)	0.64	(0.35, 1.2)	1.96	(1.3, 2.97)	1.99	(1.2, 3.29)

Note.

GHQ-12 = General Health Questionnaire – 12 items, PHQ-9 = Patient Health Questionnaire – 9 items, PC-PTSD-5 = Primary Care PTSD Screen for DSM-5, OR = odds ratio, CI = confidence interval, PPE = personal protective equipment

[a] Adjusted for gender and probable psychological distress (GHQ-12) or depression (PHQ-9) at baseline

[b] Adjusted for age and probable psychological distress (GHQ-12) or depression (PHQ-9) at baseline

[c] Adjusted for age, gender, probable psychological distress (GHQ-12) or depression (PHQ-9) at baseline, and region-level 14-day COVID-19 cumulative incidence (fixed factor)

* Baseline assessment did not include PC-PTSD-5. Models are corrected using GHQ-12 baseline total score instead

Cut-off scores used: GHQ-12 > 2, PHQ-9 > 9, and PC-PTSD-5 > 2
